# A Time Series-Based Point Estimation of Stop Signal Reaction Times: More Evidence on the Role of Reactive Inhibition-Proactive Inhibition Interplay on the SSRT Estimations †

**DOI:** 10.3390/brainsci10090598

**Published:** 2020-08-29

**Authors:** Mohsen Soltanifar, Keith Knight, Annie Dupuis, Russell Schachar, Michael Escobar

**Affiliations:** 1 Biostatistics Division, Dalla Lana School of Public Health, University of Toronto, 620, 155 College Street, Toronto, ON M5T 3M7, Canada; annie.dupuiss@mdstats.ca (A.D.); m.escobarr@utoronto.ca (M.E.); 2 The Hospital for Sick Children, Psychiatry Research, 4274, 4th Floor, Black Wing, 555 University Avenue, Toronto, ON M5G 1X8, Canada; russell.schacharr@sickkids.ca; 3 Department of Statistical Sciences, University of Toronto, 6018, Sidney Smith Hall, 100 St. George Street, Toronto, ON M5S 3G3, Canada; keithh@utstat.toronto.edu

**Keywords:** stop signal reaction times, estimation, after effects of inhibition, reactive inhibition, proactive inhibition

## Abstract

The Stop Signal Reaction Time (SSRT) is a latency measurement for the unobservable human brain stopping process, and was formulated by Logan (1994) without consideration of the nature (go/stop) of trials that precede the stop trials. Two asymptotically equivalent and larger indices of mixture SSRT and weighted SSRT were proposed in 2017 to address this issue from time in task longitudinal perspective, but estimation based on the time series perspective has still been missing in the literature. A time series-based state space estimation of SSRT was presented and it was compared with Logan 1994 SSRT over two samples of real Stop Signal Task (SST) data and the simulated SST data. The results showed that time series-based SSRT is significantly larger than Logan’s 1994 SSRT consistent with former Longitudinal-based findings. As a conclusion, SSRT indices considering the after effects of inhibition in their estimation process are larger yielding to hypothesize a larger estimates of SSRT using information on the reactive inhibition, proactive inhibition and their interplay in the SST data.

## 1. Introduction

**Figure 1:**
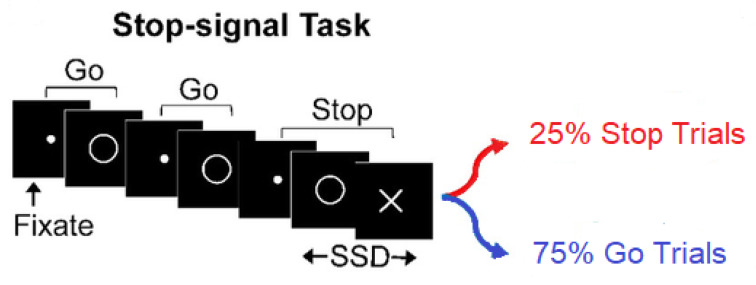
Standard Stop-signal Task (SSST) design with $p_{ss}=0.25$ including two versions of the task: 25% stop trials and 75% go trials, (Manza, P. et al., 2016 [15]-modified, permission is obtained).

Inhibitory control has theoretical and empirical importance. While its theoretical importance is rooted in its nature as an internally governed act of control, its empirical importance is due to the emergence of key results that support theories of development and inhibitory psychopathology [1]. The Stop Signal Task (SST) paradigm is a useful tool by which inhibitory control can be studied [2]. The SST has four versions: the Standard Stop Signal Task (SSST) [3,4], the Stop Signal Anticipation Task (SSAT) [5,6,7,8,9,10], the Conditional Stop Signal Task( CSST) [11,12], and the AX Continuous Performance Task(AXCPT) [13,14]. The Standard SST includes a go task and a stop task. In the go task, or “go trials”, one of two symbols, such as X or O, is presented on a computer screen. Participants are instructed to choose between the X and O as fast as possible. In the stop task, or “stop trials”, a short time after an X or O is presented, the participant hears an auditory “stop signal” through headphones; this is a Stop Signal Delay (SSD). The auditory signal indicates they must withhold their responses on that particular trial. In the most experiments, the stop trials constitute 25% of all SST trials (This is called stop-signal probability denoted by *p_ss_*. It varies depending on the SST experimenter’s objectives.) (Figure 1). There are two types of SST data: fixed SSD-based SST data and the tracking SST data. In the tracking SST data, the stop signal delay is dynamically increased by 50 ms after successful inhibition in the previous stop trial, or decreased by 50 ms after failed inhibition in the previous stop trial. Given more reliability of Stop Signal Reaction Times (SSRT) estimates in this method, it is generally recommended for Stop Signal Task experiments [2].

**Figure 2:**
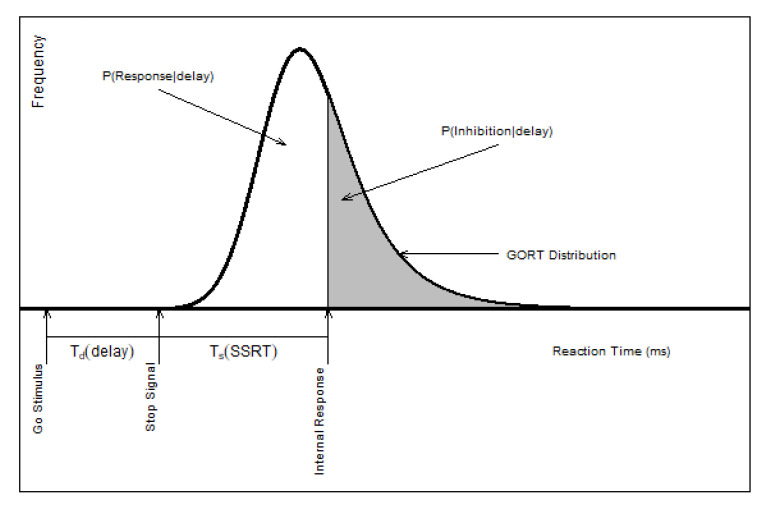
The independent horse race model: $SSD=T_{d};SSRT=T_{s}$ (Logan, 1994 [1]-modified, permission is obtained).

The independent horse race model provides a theoretical framework in which the researchers can estimate the stop process’ latency, or Stop Signal Reaction Times (SSRT), and factors associated with the probability of Successful Inhibition (SI) in the SST paradigm [16]. The independent horse race model assumes the finish times for the go reaction times (GORT) in the stop trials and the finish times for the stop process are stochastically independent. The SSRT is the time difference between the participant’s internal response and the stop signal timing (Figure 2).

Two general frequentist and Bayesian approaches have been proposed in the literature to estimate SSRT [2,17]. The frequentist approach includes three methods: Colonius’ method to calculate the entire distribution of the SSRT, the mean method to calculate the constant valued SSRT, and Logan’s 1994 integration method to calculate constant valued SSRT [17]. It has been shown that Colonius’ method is impossible to use in the human experimental context, because it requires 250,000 trials for reliable estimates [17]. Furthermore, there is higher reliability and less bias in Logan’s 1994 integration estimates versus the mean method estimates, particularly when the probability of SI is other than 50%. Given these observations, Logan’s 1994 integration method has been recommended for the estimation of SSRT [2]. For a participant with go reaction times GORT in go trials, stop signal delay SSD of $T_{d}$ and probability of successful inhibition SI in stop trials, Logan (1994) [1] proposed the following frequentist point estimation of SSRT(SSRT is indeed a function of the stop-signal probability *p_ss_*: *SSRT* = *SSRT*(*p_ss_*). Throughout this work, *p_ss_* = 0.25.):

(1) $$SSRT_{Logan1994}=Q_{GORT}\left(1-P\left(SI|\bar{T_{d}}\right)\right)-\bar{T_{d}},$$

where *Q* is the quantile function, and the average of *T_d_* is taken over all stop trials. There are two assumptions for this method: The first implicit assumption is the equal impact of go trials and stop trials in SST data; that is, the impact of the preceding trial, either go or stop, on the current stop trial SSRT estimates is assumed to be the same. The second assumption is that there is no trigger failures or the trigger failures are randomized in the SST data [18]. The authors have shown that the first assumption may be violated in the context of the tracking SST data [19]. In order to address SSRT estimation given a violated assumption of equal impact of preceding trial type on SSRT, the authors partitioned SST data to type A cluster SST data, trials following a go trial, and type B cluster SST data, trials following a stop trial. Then, by considering cluster type *GORT_A_*, *SSRT_A_*, *GORT_B_*, *SSRT_B_* and defining trial type weight *W_A_* = (#Type A stop trials)/(#Total stop trials), *W_B_* = 1 − *W_A_*, they proposed the following modified frequentist indices of SSRT [19]:

(2) $$\begin{array}{ccc} SSRT_{Weighted} & = & W_{A}\times SSRT_{A}+W_{B}\times SSRT_{B}, \end{array}$$

(3) $$\begin{array}{ccc} SSRT_{Mixture} & = & SSRT_{\left(W_{A}\times GORT_{A}+W_{B}\times GORT_{B}\right)}. \end{array}$$

It has been shown than under specific, experimenter-pre-arranged conditions, the two indices are asymptotically equivalent, given increasing number of stop trial [19]:

(4) $$|SSRT_{Weighted}-SSRT_{Mixture}|\to 0,\;\;as\;\;m_{stop}\to \infty .$$

Moreover, the authors have shown that for both cases of the real SST data and the simulated SST data [19]:

(5) $$SSRT_{Weighted}>SSRT_{Logan1994}+5.0\;ms.$$

The proposed three indices of SSRT in Equations (1)–(3) are calculated via longitudinal perspective on SST data. However, there is little information available to calculate SSRT when the SST data is considered as a time series data. The lack of literature becomes particularly important when the GORT time series has already been studied [20,21,22]. Moreover, to the authors’ best knowledge there is no study that assesses the impact of the preceding trial type (go/stop) on the current stop trial SSRT estimates in the time series context. State-Space time series models were introduced as a generalization of ordinary linear regression time series models [23,24]. They have been applied to model missing multivariate time series data of biomedical markers for cancer patients [25]. Here, the linear relationship of the current time point outcome, in terms of the previous time point outcome in the time series, and the state-space feature representated by the observation status are simultaneously useful to address the impact of the preceding trial type on the current stop trial SSRT estimates [26,27].

The primary aim of the present paper is to estimate SSRT in the context of tracking Standard SST (SSST) data, given a potential violation of the assumption of equal impact of the preceding trial type (go/stop) on the current stop trial SSRT, using the missing state-space modeling on the entire SST data set. In lights of the results of the primary goal of the work, the secondary aim is to define and formulate the external, the internal and the reactive-proactive inhibition indices, and, present a second comparison of the reactive-proactive inhibition indices and their non reactive-proactive counterparts. We hypothesize that while time series-based estimation of SSRT is significantly larger than Logan 1994 SSRT, they have equal differential impact across clinical groups such as participants with Attention Deficit Hyperactive Disorder(ADHD) and Controls. The outline of the paper is as follows. First, we consider the real SST data with given go reaction times (GORT), go reaction times on failed stop trials (SRRT), and stop signal delay time on stop trials (*T_d_*) and study disparities of internal inhibition indices. Second, in the context of time series, and using a four-stage missing data state-space modelling, we compute time series-based state space SSRT with a lognormal distributional assumption. Third, we compare the time series-based state space SSRT index with the three established indices in (1)–(3). Fourth, we repeat the previous explorations for the case of simulated data. Finally, we close with a discussion of the sensitivity of the distributional assumptions of the missing data state-space modeling, and the asymptotic behavior of the disparities between these indices.

## 2. Methods and Materials

### 2.1. Data

The study data included two sets of the real data and simulated data described below.

#### 2.1.1. The Real Data

Data was collected at the Ontario Science Center in Toronto, Canada from 2009 to 2010. The sample includes 16,099 participants, aged 6 to 17 years old [28]. Self or parent-reported demographic data were obtained. The required consent for the SST experiment was given by the participants’ parents. Each participant completed a stop signal task (SST) comprised of 5 blocks of 24 trials (one practice block and four main blocks). Within the blocks, 25% of the trials contained a stop signal. The tracking stop signal task algorithm was designed so that the probability of successful inhibition converged on 50%. The study’s variables included stop-signal delay (centi-seconds), previous trial type (stop/go), and ADHD status (yes/no). The ADHD variable was defined based on SWAN z-score [29], whether the score falls in the top 10% of the distribution (defined as ADHD case) or not (defined as control case).

#### 2.1.2. The Simulated Data

The simulations are based on the assumption of the independent race model with features in Table 1. Independent GORT and SSRT via the tracking method with initial *SSD* = 200 ms were simulated (see Appendix A). On the stop trials, a successful inhibition was considered one for which *GORT* > *SSRT* + *SSD*; and, otherwise, it was considered a failed inhibition. An ex-Gaussian distribution was assumed for GORT, SSRT, and it was simulated by R package GAMLSS [30]. This distribution has been extensively used in psychology, neuroscience, and as a time model for cognitive process in the study of reaction times [31,32,33]. We simulated SST data for each participant with type A GORT and SSRT ex-Gaussian distributions, type B GORT and SSRT ex-Gaussian distributions, and weights *W_A_* = 0.75 and *W_B_* = 0.25 (see Appendix B). We randomly merged the two cluster A and cluster B SST data for each participant. The baseline ex-Gaussian distributional forms for type A cluster, type B cluster SST data, and participant SST sample are given in Table 1.

### 2.2. Participants

Participants were partitioned to the real data and the simulated data as follows:

For the real data, a random subsample of 44 participants (11 ADHD; 33 Control) with mean age of 10.3 years (ADHD), 12.7 years (Control), with 96 SST trials, and with 10, 11, 12, 13, 14 stop trials preceded by a stop trial (thus, 14, 13, 12, 11, 10 stop trials preceded by a go trial) was selected from the real SST data in Section 2.1.1. This choice of subsample was justified in order to have close number of stop trials of each type (e.g., 10 vs. 14; 11 vs. 13; 12 vs. 12; 13 vs. 11; 14 vs. 10), and consequently, to reduce the bias in the estimations. Finally, the violation of the assumption of equal impact of the preceding trial type (go/stop) on the SSRT was assessed in analysis as it follows in the results Section 3.1.

For the simulated data, 44 participants with a variety of increasing or decreasing mean and variance of their underlying SSRT distributions were simulated. Each simulated participant had 96, 192, 288, 384, 480, and 960 SST trials. Finally, the simulated type A SSRT and type B SSRT all violated the assumption of equal impact of the preceding trial type (go/stop) on the SSRT. Indeed, in each category of simulations, the mean of type A SSRT is 20 ms smaller than that of type B SSRT.

### 2.3. Statistical Inference

#### 2.3.1. Time Series Based State- Space SSRT (*SSRT*_*SS.Logan*1994_)

**Figure 3:**
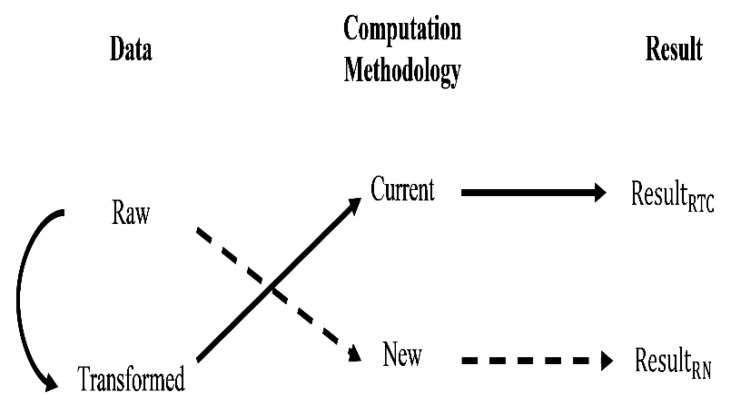
A general framework for data and statistical methodology to conclude the new estimation results (dashed path: process for $SSRT_{Weighted},SSRT_{Mixture}$; solid path: process for time series-based $SSRT_{SS.Logan1994}$).

There are two perspectives to develop new statistical estimations (Figure 3): First, we develop the new statistical methodology and apply it to the available raw data for measuring the quantities or statistics of interest (Figure 3: dashed path). An example of such method was presented in [19] for the $SSRT_{Weighted}$ and $SSRT_{Mixture}$ indices. Second, we enrich and transform the available raw data, and apply the current statistical methodology on the transformed data to measure the quantities or statistics of interest (Figure 3: solid path). The example of this method is presented here in this paper for the case of time series-based SSRT denoted by $SSRT_{SS.Logan1994}$.

**Figure 4:**
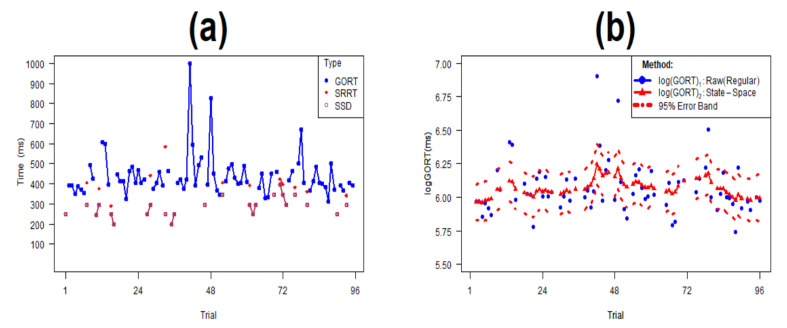
(**a**) 3-variables time series plot of the Standard Stop Signal Task (SSST) data; (**b**) Estimation of time series-based state-space log (GORT) for the GORT data in the panel (**a**).

Our proposed time series-based SSRT estimation method (Figure 3: solid path) is compromised of the following four steps:

   **Step (i):** We assumed a lognormal parametric distribution for the raw SST time series data $x_{t1}^{*}=GORT_{t},x_{t2}^{*}=SRRT_{t},$ and $x_{t3}^{*}=SSD_{t}$ (Figure 4a) and, then we considered the normal input raw SST data $x_{t1}=log\left(x_{t1}^{*}\right),x_{t2}=log\left(x_{t2}^{*}\right)$ and $x_{t3}=log\left(x_{t3}^{*}\right)$. This transformation is due to the fact that the state–space modelling of time series requires the input data to have normal distribution [34]. In addition, the original lognormal distributional assumption for Reaction Times (RT) data is among the most accepted distributional forms in the RT literature, and it removes considerable skewness, making the data’s distribution near normal [35].

**Step (ii):** We fit a Frequentist missing state-space model using Expectation Maximization (EM) algorithm with relative tolerance of 1% by R package ASTSA [27,36] to the incoming raw SST data in Step (i) as in Equation (6). Here, ${\left(x_{t}\right)}_{3\times 1}={\left(x_{t1},x_{t2},x_{t3}\right)}^{t},$ and the observation matrix $A_{t}^{\left(1\right)}$ carries the impact of the trial type information from the previous trial to the current trial. It is either identity matrix or partial identity matrix with some diagonal values of 0 whenever the preceding trial type is “Go”.

(6) $$\begin{array}{ccc} \mathrm{State}\;\mathrm{Equation}: & {\left(x_{t}\right)}_{3\times 1}={\left(\Phi \right)}_{3\times 3}.{\left(x_{t-1}\right)}_{3\times 1}+{\left(w_{t}\right)}_{3\times 1}: & w_{t}{∼}^{iid}N\left(0_{3\times 1},Q_{3\times 3}\right)\\ \mathrm{Observation}\;\mathrm{Equation}: & {\left(y_{t}^{\left(1\right)}\right)}_{q1t\times 1}={\left(A_{t}^{\left(1\right)}\right)}_{q1t\times 3}.{\left(x_{t}\right)}_{3\times 1}+{\left(v_{t}^{\left(1\right)}\right)}_{q\times 1}: & v_{t}^{\left(1\right)}{∼}^{iid}N\left(0_{q1t\times 1},R_{q1t\times q1t}\right) \end{array}$$

**Step (iii).** We used the estimated output $\hat{x_{t1}}=\hat{logGORT_{t}}(1\leq t\leq m\left(m=72k\;\left(k=1,2,3,4,5,10\right)\right)$ in Equation (6) in Step (ii) and the frequentist MLE methods to fit normal distributions $N(\hat{\mu },\hat{\sigma^{2}})$ to the corresponding state- space SST data (72k GORTs, 8k−14k SRRTs, 24k SSDs: k=1,2,3,4,5,10) matched to the original SST data (Figure 4b).

**Step (iv).** The time series-based state-space estimation of SSRT for given probability of successful inhibition P(SI) was computed as in (7) using parameters estimations $\left(\hat{\mu },\hat{\sigma }\right)$ in Step (iii) where $\Phi^{-1}$ is the quantile function of standard normal distribution, and the average of $T_{d}$ is taken over all matched state-space stop trials:

(7) $$SSRT_{SS.Logan1994}=exp\left(\hat{\mu }+\hat{\sigma }.\Phi^{-1}\left(1-P\left(SI\right)\right)\right)-\bar{T_{d}},$$

Table 2 compares the overall methodology applied in calculations of SSRT indices (1)–(3) with that of SSRT index in (7) with repeating steps (i)–(iv) with normal assumptions for the input raw SST data:

#### 2.3.2. Inhibition Indices and Hypothesis Tests

*Inhibition Indices*

We consider three types of inhibition indices for the proactive inhibition (anticipation of stopping) and reactive inhibition (outright stopping) including external indices, internal indices and reactive-proactive indices. We applied the later two definitions in this work:

**Definition 1** (**external inhibition index**)**.**  *The external inhibition index is the one calculated based on the external manipulation of stop signal probability*$p_{SS}$*levels in the Stop Signal Task paradigm. The external proactive inhibition index is calculated based on the difference (or a variation of the differences) of GORTs in the associated arms of the SST paradigm [3,5,6,7,8,9,37,38]. For example,*$\Delta GORT=GORT_{p_{ss}=0.50}-GORT_{p_{ss}=0.25}$*. The external reactive inhibition index is quantified based on the stop signal reaction times for the stop trials arm of the SST paradigm [3,5,6,7,8,9,37,38]. For example,*$\Delta SSRT=SSRT_{p_{ss}=0.50}-SSRT_{p_{ss}=0.25}$.

**Definition 2** (**internal inhibition index**)**.**  *The internal inhibition index is the one computed based on the previous trial type (go/stop) for a given fixed stop signal probability* $p_{SS}$ *(e.g., 0.25) in the Stop Signal Task paradigm. The internal proactive inhibition index is calculated based on the difference (or a variation of the differences) of GORTs in the associated arms of the SST paradigm [19]. For example,* $\Delta GORT=GORT_{B}-GORT_{A}$ *(Type A GORT (* $GORT_{A}$ *) is for the trial following a go trial. Type B GORT (* $GORT_{B}$ *) is for the trial following a stop trial.). The internal reactive inhibition index is quantified based on the stop signal reaction times for the stop trials arm of the SST paradigm [19]. For example,* $\Delta SSRT=SSRT_{B}-SSRT_{A}$ *(Type A SSRT (* $SSRT_{A}$ *) is for the trial following a go trial. Type B SSRT (* $SSRT_{B}$ *) is for the trial following a stop trial.).*

**Definition 3** (**reactive-proactive inhibition index**)**.** *The reactive-proactive inhibition index is the inhibition quantity considering the reactive-proactive interplay in its estimations [19]. There are two such indices:* $GORT_{Reactive-Proactive}$ *and* $SSRT_{Reactive-Proactive}.$ *An example of the former index is* $W_{A}.GORT_{A}+W_{B}.GORT_{B}.$ *Examples of the later index include* $SSRT_{Mixture},SSRT_{Weighted}$ *and* $SSRT_{SS.Logan1994}.$ *Two examples of the non reactive-proactive inhibition index are* GORT *and* $SSRT_{Logan1994}.$

*Hypothesis Tests*

Three sets of hypothesis tests were conducted including those for internal inhibition indices, those between reactive-proactive inhibition indices and their non reactive-proactive counterparts, and, those for reactive-proactive inhibition indices across clinical groups (ADHD vs. Controls).

First, for the case of internal(trial by trial) based comparisons, the following hypothesis tests were conducted for fixed stop signal probability (0.25):

(8) $$\begin{array}{ccc} & & H_{1}:GORT_{B}\neq GORT_{A}\;\;\;vs.\;\;\;H_{0}:GORT_{B}=GORT_{A}, \end{array}$$

(9) $$\begin{array}{ccc} & & H_{1}:SSRT_{B}\neq SSRT_{A}\;\;\;vs.\;\;\;H_{0}:SSRT_{B}=SSRT_{A}. \end{array}$$

Second, for the case of comparison of estimations, the following statistical hypothesis test is conducted:

(10) $$\begin{array}{ccc} & & H_{1}:SSRT_{SS.Logan1994}\neq SSRT_{Logan1994}\;\;\;vs.\;\;\;H_{0}:SSRT_{SS.Logan1994}=SSRT_{Logan1994}. \end{array}$$

Third, for the case of comparison of the differential impact between clinical groups, the following statistical hypothesis test is conducted (A useful index of SSRT is naturally expected to report similar or larger differential estimations between clinical groups (e.g., ADHD vs. Control) compared to the available SSRT indices. This characterizes it as a new potential biomarker for the participants’ clinical status in other studies [3,4,9,39]):

(11) $$\begin{array}{ccc} & & H_{1}:SSRT_{SS.Logan1994}^{ADHD}\neq SSRT_{SS.Logan1994}^{Control}\;\;\;vs.\;\;\;H_{0}:SSRT_{SS.Logan1994}^{ADHD}=SSRT_{SS.Logan1994}^{Control}. \end{array}$$

Similar hypothesis tests are conducted with replacing $SSRT_{Logan1994}$ in hypothesis test (10) with $SSRT_{Mixture}$ and $SSRT_{Weighted};$ and, with replacing $SSRT_{SS.Logan1994}$ in the hypothesis test (11) with $SSRT_{Mixture}$ and $SSRT_{Weighted}.$

All formerly established SSRT indices in Equations (1)–(3) were compared with the time series-based index in Equation (7) using the paired *t*-tests (An ideal reporting statistics here is the Linear Mixed Model (LMM) effects. However, given no additional covariate in the analysis and equvalency of the varying intercept LMM and the paired *t*-test [40] we considered the later one.) (PROC TTEST, [41]). Given the distributional assumption for the SST data in the state space modelling (three variate lognormal or normal), independent sample *t*-tests were conducted between ADHD and control groups within each SSRT index.

## 3. Results

The results are divided into three subsections. In Section 3.1, a moderate evidence is presented on the potential disparity for the proactive internal inhibition index SSRT and the need to consider the reactive inhibition-proactive inhibition interplay in the SSRt estimations. Next, in Section 3.2, the time series-based state space SSRT index is compared with $SSRT_{Weighted}$,$SSRT_{Mixture}$, and $SSRT_{Logan1994}$ in terms of size and differential impact between clinical groups. Finally, in Section 3.3, the comparisons in terms of size of the estimates are repeated for the simulated data and the asymptotic behaviour of the sizes. Also, their sensitivity to the distributional assumptions is studied.

### 3.1. Disparities of the Internal Inhibition Indices

This section deals with calculation and comparisons of internal inhibition indices inspired by trial by trial approach proposed in ([10,19]). Table 3 presents the comparison results for proactive index Δ*GORT* and reactive index *SSRT*:

There are three key observations: First, there is significant effect of 71.6 ms in the internal proactive inhibition (95%CI = (51.4, 91.8)). Second, the internal reactive inhibition indices $SSRT_{A}$ and $SSRT_{B}$ are significantly different than $SSRT_{Logan1994}.$ Third, the finding on the positive non significant difference between $SSRT_{A}$ and $SSRT_{B}$ is consistent with the results reported by internal reactive inhibition index in [19] in terms of effect size, but, it is inconsistent in terms of significancy of the effect. However, given (i) disparities of both trial type SSRTs with that of Logan 1994 SSRT, and (ii) magnitude of the disparity between $SSRT_{A}$ and $SSRT_{B}$ (i.e., 8.8 ms), and (iii) the fitted non-identity linear regression line $SSRT_{Bi}=\beta_{0}+\beta_{1}.\;SSRT_{Ai}+\epsilon_{i}:\epsilon_{i}∼N\left(0,\sigma_{e}^{2}\right),\beta_{0}\;=\;96.2$, (95%CI = (4.0, 188.4)); $\beta_{1}\;=\;0.53$ (95%CI = (0.06, 1.0)), there exists a moderate evidence of violation of the assumption of equal impact of the preceding trial type (go/stop) on the current stop trial SSRT leading to SSRT disparities across clusters of SST data.

In the next section, we propose a time series-based index of SSRT based on reactive inhibition-proactive inhibition interplay addressing the internal effect of the previous trial type in the SSRT estimations.

### 3.2. Time Series Based State-Space SSRT for the Real SST Data

This section deals with the overall time series-based state space estimation of SSRT from internal (trial by trial) perspective considering the reactive inhibition-proactive inhibition interplay in the SSRT estimation process. Table 4 presents results for the time series-based estimated state-space SSRT and compares it to the established SSRTs.

**Figure 5:**
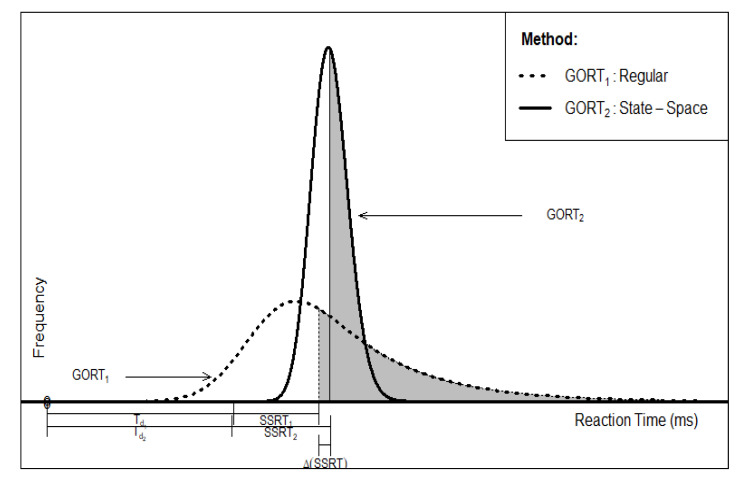
Regular and time series-based State-Space estimations of SSRT.

There are five key results from Table 4. First, the $SSRT_{SS.Logan1994}$ was significantly larger than $SSRT_{Logan1994}$ under both distributional assumptions (Lognormal: 13.1: 95%CI = (8.1, 17.6); Normal: 21.4: 95%CI = (17.4, 26.3)); second, there were no significant differences between $SSRT_{SS.Logan1994}$ and two former indices $SSRT_{Mixture}$ and $SSRT_{Weighted}$ overall, under the lognormal distributional assumption. However, under normal distributional assumption, the former index was significantly larger than the latter two [8.3:95%CI=(0.2,16.4);7.7:95%CI=(1.3,14.1),respectively]. Third, the ADHD participants had 58.6 ms [95%CI=(3.0,114.2)] higher $SSRT_{SS.Logan1994}$ values than controls under the lognormal distributional assumption. A similar result was observed under the normal distributional assumption. Fourth, the differential impact of $SSRT_{SS.Logan1994}$ was more or less similar the two former modified indices $SSRT_{Mixture}$ and $SSRT_{Weighted}$ under the lognormal distributional assumption (58.6 ms vs. 65.6 ms, 62.3 ms with overlapping 95%CI). Finally, similar conclusions were found under the normal distributional assumption. Figure 5 depicts the comparison of the regular estimation of SSRT given by (1) and its state-space counterpart given by (7), given by the results in Table 4. From Table 4a, it follows that under different underlying distributional assumptions for GORT, SRRT, and SSD, we obtain different estimates when comparing $SSRT_{SS.Logan1994}$ and the other three indices. To confirm the assumption of sensitivity of the state-space SSRT estimation to the underlying distributional assumption, and, adjust for potential confounders in the estimations [42], we will conduct the comparisons for the simulated data in the next section.

### 3.3. Simulations and Asymptotic Behaviour

This section deals with calculation and comparison of the reactive-proactive SSRT indices and their non reactive-proactive counterparts for the simulated SST data as shown in Section 2.1.2. The aim is to check the impact of the underlying distributional assumptions in the state-space models on the estimated SSRT indices and to assess the potential confounding effects of the number of SST trials, the clinical status and the age in the disparities of the mentioned SSRT indices. Table 5 presents the results of pairwise *t*-tests:

The presented results are for each given participant sample size m under the lognormal distributional assumption for the simulated ex-Gaussian GORT, SRRT, and SSRT. Such an assumption is justified, given that the shifted lognormal distribution provides a good fit for the ex-Gaussian distribution of the RT data [43]. Comparing the results from Table 4, Panel(a), and Table 5, we conclude:

Under the lognormal assumption for the real data:

Result (i): The difference between the $SSRT_{SS.Logan1994}$ and $SSRT_{Logan1994}$ in the simulated data is the same as in the original real data. However, the size of differences in the former (8.1–11.8 ms) is smaller than the latter (13.1 ms), and with increasing simulated sample sizes, their gap diminishes.
Result (ii): The difference between $SSRT_{SS.Logan1994}$ and $SSRT_{Mixture}$ in the simulated data is in the range 8.5–11.7 ms and very different from the non-significant difference in the original real data.
Result (iii): The difference between $SSRT_{SS.Logan1994}$ and $SSRT_{Weighted}$ in the simulated data is in the range of 4.0–5.4 ms, and somewhat different than that of their non-significant difference in real data.

Under the normal assumption for the real data:

Result (i): The difference between $SSRT_{SS.Logan1994}$ and $SSRT_{Logan1994}$ in the simulated data (8.1 ms–11.8 ms) is significantly smaller than in the original real data (21.9).
Result (ii): The difference between the $SSRT_{SS.Logan1994}$ and $SSRT_{Mixture}$ in the simulated data (8.1 ms–11.8 ms) is similar to that of the real data (8.3 ms).
Result (iii): The difference between $SSRT_{SS.Logan1994}$ and $SSRT_{Weighted}$ in the simulated data (8.1 ms–11.8 ms) is similar to that of real data (7.7 ms).

**Figure 6:**
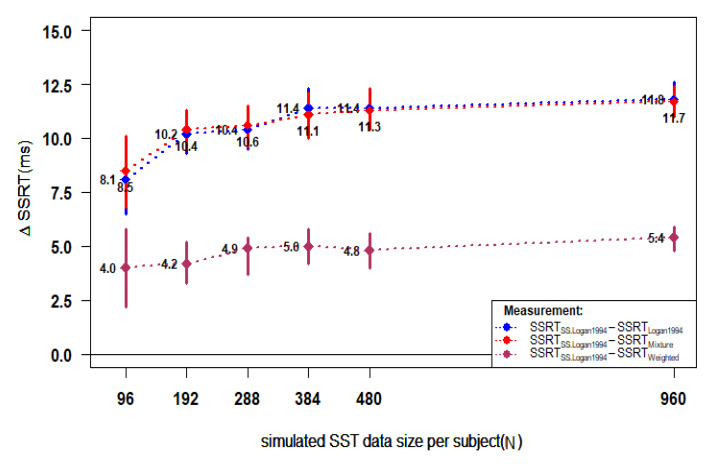
Disparities of simulated SSRT indices by sample size (n = 264).

These two sets of results show that one needs to check underlying distributional assumptions for state-space models in calculating $SSRT_{SS.Logan1994}$. Figure 6 presents the indices’ differences in terms of simulated sample size. There are two main results: First, the main increment on the index difference occurs from sample size m=96 to m=192; Second, after simulated sample size m=480, the trend is almost asymptotically constant.

## 4. Discussion

This study has presented a time series-based methodological approach for a more informed estimation of SSRT by considering state-space nature of SST time series data, and lognormal format of the involved distributions offering a time series-based index of SSRT. It introduced time series=based state–space SSRT as the third index of SSRT, which considered trial order in the stop task data. It hypothesized that considering previous trial type (stop/go) in calculation of the time series-based index does impact estimations of SSRT, as it was shown in the case of the longitudinal approach [19].

The majority of findings of the study affirmed the hypothesis. State-space estimations of Logan’s 1994 SSRT were 13.1 ms and 21.9 ms significantly larger than their regular estimations under a normal or lognormal distribution assumption for SST data, respectively. This result was confirmed by similar significantly higher estimations (8.1 ms–11.8 ms) from simulated SST data under the ex-Gaussian distributional assumption. However, there were no significant differences between two indices of SSRT on their differential impact between clinical groups. There was consistency in comparing the results of the state-space SSRT and the Logan 1994 SSRT indices using both real and simulated SST data. While in the majority of cases, the time series-based index is different from established indices (as shown in this study), there are special cases where these four indices will be precisely equal. Two special cases include when each stop trial is preceded by a go trial, i.e.,  ${\left(A_{t}^{\left(1\right)}\right)}_{q1t\times 3}=0_{3}$ and when each stop trial is preceded by another stop trial, i.e.,  ${\left(A_{t}^{\left(1\right)}\right)}_{q1t\times 3}=I_{3}.$

The study’s results based on the time series method were consistent with those of longitudinal method [19], considering the impact of the preceding trial type on the current stop trial SSRT in the calculation of SSRT. The first consistency is that when the researcher considers the trial order when considering the SST data, they obtain significantly larger estimates versus when they ignore trial order. This is the common conclusion in both approaches. One explanation for this commonality is that a participant’s stopping skills improves immediately after stop trials compared to go trials in the SST. This is in accord with examples in previous literature, such as a participant’s optimized control skills in playing video games with dual task and task switching situation [44], and a participant’s improved visuomotor control in playing action video games [45]. In our case, once the order of SST trials is considered in the calculations, for the those preceded by a stop trial the participant’s stopping improves by his or her on taking longer go reaction times (GORT), and hence, the latency of the stopping process SSRT increases. This yields to increase in the estimated SSRT. The second consistency is that there is no statistically significant difference between clinically differential impact(ADHD versus Control) using longitudinal perspective estimations of SSRT versus time series-based estimation of SSRT. On defining the ADHD based on SWAN z-score we followed a trait based approach rather than emulating diagnosis.

The study’s methodology paves the way to hypothesize: (i) the estimations of SSRT based on the interplay between reactive inhibition and proactive inhibition, and (ii) their relationship with those estimations of SSRT ignoring such interplay information. Combined with findings in [19], it presented additional evidence on the mitigating role of the reactive inhibition-proactive inhibition interplay on the reactive inhibition manifested by the higher SSRT values, which is for two special cases:

(12) $$\begin{array}{ccc} SSRT_{Reactive-Proactive} & > & SSRT_{Non.Reactive-Proactive}. \end{array}$$

These findings were in the context of Standard Stop Signal Task (SSST), internal perspective on inhibition indices and through reactive-proactive SSRT index comparisons with their non reactive-proactive counterparts. The advantages included (i) simpler SSST design (compared to SSAT, CSST, or AXCPT designs), and (ii) more precise estimations of SSRT (compared to non reactive-proactive). However, the key disadvantage is more complex calculations of SSRT.

The study’s proposed time series-based method is, however, less favourable than that of time in task longitudinal-based method [19] given a few considerations as follows: First, the calculations in the proposed method (in particular compared to the Weighted SSRT index in the old method) are more difficult than the old one. Second, the calculations in the method are susceptible to satisfaction of underlying lognormal distributional assumption for the SST data. Third, the calculations in the method are dependent to the size of the relative tolerance of the missing data EM algorithm. Finally, given that (i) ADHD as a trait is likely to reflect participants who make alot of errors in go trials and many signal responds in stop trials; (ii) the proposed estimation method considered preceding trial type into account; there was no progress in finding better differential impact (ADHD versus Control) in the proposed method.

This study’s findings are restricted in a few aspects. The first is the assumption of a lognormal distribution for GORT, SRRT, and SSRT in the state–space estimations of Logan 1994 SSRT. The optimum situation would be ex-Gaussian distributional assumption for GORT, SRRT, and SSRT in the new method such that the only remaining difference between the conventional method and the new method would be consideration of the nature (go/stop) preceding trials in the SST data. Thus, such assumption limited comparison of the results in their more customary assumption of ex-Gaussian distribution [2]. The second is the sensitivity of the state-state approach in calculating SSRT to a multivariate normal distributional assumption of the SST data, and upon violation of normality, inconsistent results may yield. This is evident from both real and the simulated SST data results, in simulated data, given good fit of the lognormal distribution to the simulated Ex-Gaussian SST data, the results for the calculated state-space SSRT versus the regular SSRT are consistent. The third one is that state-space calculation of SSRT depends on the relative tolerance of the missing data EM algorithm in the calculation of the state-space SST data. While we chose 1% for this purpose, other values may yield different state-space SST data and a different state-space SSRT estimate. Consequently, they may impact their comparison of non-state space SSRT estimates. Finally, for simplicity of the calculations, it was assumed that there were no trigger failures or randomized trigger failures in the SST data [18].

The approach outlined in this study should be replicated in the future research in three directions. The first is that the study should be replicated for in adult participants who can perform longer tasks and produce a higher number of stop trials (e.g., 200 SST trials with 50 stop trials, as recommended [2], to confirm the current results at older ages and across a larger number of trials. The second is to consider non randomized trigger failures and their probabilities in the SST data in the calculations of the state-space Logan 1994 SSRT and to compare the results with the former established estimates. Finally, it remains for further work to examine the general validity of Inequality (12) as the following hypothesis test (13) when considering the information on reactive inhibition, proactive inhibition and their interplay in the estimations of SSRT(The test (13) is conducted for a given fixed stop signal probability $p_{ss}$ (e.g., 0.25) in (0,1).):

(13) $$\begin{array}{ccc} & & H_{1}:SSRT_{Reactive-Proactive}>SSRT_{Non.Reactive-Proactive}\;\;\;vs.\\ & & H_{0}:SSRT_{Reactive-Proactive}=SSRT_{Non.Reactive-Proactive}. \end{array}$$

## 5. **Conclusions**

The trial type (go/stop) in the entire stop signal task data was shown to be a key factor in the estimation of the associated SSRT based on the time in task longitudinal method [19]. This study provided further evidence on this finding from time series perspective, paving the way for more refinement in the estimates. Given consistency of results in both methods and advantages of the first method, the researchers are recommended to consider Weighted SSRT ($SSRT_{Weighted}$) as the latest optimum option for the point estimation of the latency stopping process in the brain and a starting point to estimate it based on the the interplay between reactive inhibition and proactive inhibition.

## Figures and Tables

**Table 1:** Structure of simulated stop task data by participant sample size (SST data sample size per participant: N = 96,192,288,384,480,960).

SSRT (Δ Mean, Δ STD)	n (Participants)	Cluster Type	n (Participants): Cluster	GORT Distribution	SSRT Distribution
(increasing, increasing)	11	A	11 (a=2k:1≤k≤11)	ExG (300,35,30)	ExG (130+a,70+a,60+a)
		B	11 (a=2k:1≤k≤11)	ExG (450,50,30)	ExG (150+a,90+a,60+a)
(increasing, decreasing)	11	A	11 (a=2k:1≤k≤11)	ExG (300,35,30)	ExG (130+a,70−a,60)
		B	11 (a=2k:1≤k≤11)	ExG (450,50,30)	ExG (150+a,90−a,60)
(decreasing, increasing)	11	A	11 (a=2k:1≤k≤11)	ExG (300,35,30)	ExG (130−a,70+a,60)
		B	11 (a=2k:1≤k≤11)	ExG (450,50,30)	ExG (150−a,90+a,60)
(decreasing, decreasing)	11	A	11 (a=2k:1≤k≤11)	ExG (300,35,30)	ExG (130−a,70−a,60−a)
		B	11 (a=2k:1≤k≤11)	ExG (450,50,30)	ExG (150−a,90−a,60−a)

**Table 2:** Comparison of the time series-based and other methods of estimations of SSRT.

Figure 3 (dashed path)	$SSRT_{Weighted},SSRT_{Mixture}$	Used Data	Raw GORT and SSD
		Methodology	No impact of the preceding trial on the current trial
		Distribution	Ex-Gaussian
Figure 3 (solid path)	$SSRT_{SS.Logan1994}$	Used Data	Estimated state-space GORT and SSD
		Methodology	Impact of the preceding trial on the current trial
		Distribution	Lognormal/Normal

**Table 3:** Paired *t*-test results of the internal reactive and proactive inhibition indices for Standard Stop Signal Task (SSST) with $p_{ss}=0.25$ (n = 44).

Inhibition Type	Index Disparities	Mean (95%CI)	t	Sig. (2-Tailed)
Proactive	$\Delta GORT=GORT_{B}-GORT_{A}$	71.6 (51.4, 91.8)	7.2	<0.0001
Reactive	$\Delta SSRT=SSRT_{Logan1994}-SSRT_{A}$	−10.3 (−20.6, 0.0)	−2.0	0.05
Reactive	$\Delta SSRT=SSRT_{Logan1994}-SSRT_{B}$	−19.1 (−39.6, −1.5)	−1.9	0.06
Reactive	$\Delta SSRT=SSRT_{B}-SSRT_{A}$	8.8 (−20.1, 37.7)	0.6	0.5 *

* See the subsequent analysis with non-identity linear regression line estimations.

**Table 4:** Paired *t*-test and two sample *t*-test results of SSRT indices by distributional assumption (n = 44).

**(a) Measurement Comparisons**					
**Measurement**	**Population**	**Distribution**	**Mean (95%CI)**	***t***	**Sig. (2-Tailed)**
$SSRT_{SS.Logan1994}-SSRT_{Logan1994}$	Overall	Lognormal	13.1 (8.4, 17.6)	5.7	<0.0001
		Normal	21.9 (17.4, 26.3)	9.9	<0.0001
$SSRT_{SS.Logan1994}-SSRT_{Mixture}$	Overall	Lognormal	−0.6 (−9.8, 8.7)	−0.1	0.9
		Normal	8.3 (0.2, 16.4)	2.1	0.04
$SSRT_{SS.Logan1994}-SSRT_{Weighted}$	Overall	Lognormal	−1.2 (−8.4, 6.0)	−0.3	0.7
		Normal	7.7 (1.3, 14.1)	2.4	0.02
**(b) Differential Impact**					
**Measurement**	**Population**	**SST Distribution**	**Mean (95%CI)**	***t***	**Sig. (2-tailed)**
$SSRT_{SS.Logan}$	ADHD vs. Control	Lognormal	58.6 (3.0, 114.2)	2.3	0.04
		Normal	58.8 (1.8, 115.6)	2.3	0.04
$SSRT_{Logan1994}$		Ex-Gaussian	66.5 (10.5, 122.5)	2.6	0.02
$SSRT_{Mixture}$		Ex-Gaussian	65.6 (5.4, 125.9)	2.4	0.04
$SSRT_{Weightd}$		Ex-Gaussian	62.3 (5.1, 119.5)	2.4	0.04

**Table 5:** Paired *t*-test results of comparison between simulated time series-based state-space SSRT index and non time series-based counterparts by m (n = 264).

Pair	N(#SST)	m (#stop)	Mean (95%CI)	*t*	Sig. (2-Tailed)
$SSRT_{SS.Logan1994}-SSRT_{Logan1994}$			8.1(6.5,9.8)	9.9	<0.0001
$SSRT_{SS.Logan1994}-SSRT_{Mixture}$	96	24	8.5(6.8,10.1)	10.3	<0.0001
$SSRT_{SS.Logan1994}-SSRT_{Weighted}$			4.0(2.2,5.8)	4.5	<0.0001
$SSRT_{SS.Logan1994}-SSRT_{Logan1994}$			10.2(9.3,11.1)	23.1	<0.0001
$SSRT_{SS.Logan1994}-SSRT_{Mixture}$	192	48	10.4(9.5,11.3)	23.1	<0.0001
$SSRT_{SS.Logan1994}-SSRT_{Weighted}$			4.2(3.3,5.2)	8.8	<0.0001
$SSRT_{SS.Logan1994}-SSRT_{Logan1994}$			10.4(9.5,11.2)	22.8	<0.0001
$SSRT_{SS.Logan1994}-SSRT_{Mixture}$	288	72	10.6(9.6,11.5)	22.8	<0.0001
$SSRT_{SS.Logan1994}-SSRT_{Weighted}$			4.9(3.7,5.4)	11.7	<0.0001
$SSRT_{SS.Logan1994}-SSRT_{Logan1994}$			11.4(10.4,12.3)	24.5	<0.0001
$SSRT_{SS.Logan1994}-SSRT_{Mixture}$	384	96	11.1(10.0,12.1)	12.3	<0.0001
$SSRT_{SS.Logan1994}-SSRT_{Weighted}$			5.0(4.2,5.8)	12.3	<0.0001
$SSRT_{SS.Logan1994}-SSRT_{Logan1994}$			11.4(10.5,12.3)	26.6	<0.0001
$SSRT_{SS.Logan1994}-SSRT_{Mixture}$	480	120	11.3(10.4,12.3)	24.1	<0.0001
$SSRT_{SS.Logan1994}-SSRT_{Weighted}$			4.8(4.0,5.6)	12.4	<0.0001
$SSRT_{SS.Logan1994}-SSRT_{Logan1994}$			11.8(11.2,12.6)	34.0	<0.0001
$SSRT_{SS.Logan1994}-SSRT_{Mixture}$	960	240	11.7(11.0,12.4)	33.3	<0.0001
$SSRT_{SS.Logan1994}-SSRT_{Weighted}$			5.4(4.8,5.9)	20.0	<0.0001

## Abbreviations

The following abbreviations are used in this manuscript:

ADHD	Attention Deficit Hyperactivity Disorder
AXCPT	AX Continuous Performance Task
CSST	Conditional Stop Signal Task
EM	Expectation Maximization
GORT	Reaction time in a go trial
GORTA	Reaction time in a type A go trial
GORTB	Reaction time in a type B go trial
LMM	Linear Mixed Model
RT	Reaction Times
SI	Successful Inhibition
SR	Signal Respond
SRRT	Reaction time in a failed stop trial
SSAT	Stop Signal Anticipation Task
SSD	Stop Signal Delay
SSRT	Stop Signal Reaction Times in a stop trial
SSRTA	Stop Signal Reaction Times in type A stop trial
SSRTB	Stop Signal Reaction Times in type B stop trial
*SSRT*_*Logan*1994_	Logan 1994 SSRT
*SSRT_Mixture_*	Mixture SSRT
*SSRT*_*SS.Logan*1994_	Time Series-based State-Space SSRT
*SSRT_Weighted_*	Weighted SSRT
SSST	Standard Stop Signal Task
SST	Stop Signal Task
SWAN	Strengths and Weakness of ADHD-symptoms and Normal behavior rating scale

## Appendix A. R Software Simulation Code of Stop Signal Task Data with Tracking Method

We use R software(R version 4.0.0 (2020-04-24)) to simulate a data set of 200 observations with 160 go trials and 40 stop trials with initial SSD = 200 ms. The GORT has Ex-Gaussian distribution with parameters *μ* = 350, *σ* = 50, *τ* = 30, and the SSRT has Ex-Gaussian distribution with parameters *μ* = 100, *σ* = 70, *τ* = 60.

###Begin

###Call Required Libraries

library(gamlss.dist)

library(gamlss)

library(goftest)

library(retimes)

require(gamlss.dist)

require(gamlss)

require(retimes)

###Set up the Simulation Parameters

#Total number of Trials#

n=200

#Total number of stops#

m=40

mu.go=350

sigma.go=50

nu.go=30

mu.SSRT=100

sigma.SSRT=70

nu.SSRT=60

SRRT0=-999

###Set up the Simulation Data set

id<-as.vector(matrix(1,nrow=1,ncol = n))

index<-as.vector(matrix(0,nrow=1,ncol = n))

#This is for the stop trial checking whether to add 50 to SSD for the next one#

GORT <- round( rexGAUS(n, mu = mu.go , sigma = sigma.go , nu = nu.go), digits = 1)

SSRT <- round( rexGAUS(n, mu = mu.SSRT , sigma = sigma.SSRT , nu = nu.SSRT), digits = 1)

SSD<-as.vector(matrix(0,nrow=1,ncol = n))

SRRT<-as.vector(matrix(SRRT0,nrow=1,ncol=n))

SSD[1]<- 200

TrialType <-as.vector(matrix(’Go’,nrow=1,ncol = n))

Inhibition <-as.vector(matrix(’-999’,nrow=1,ncol = n))

###Run Simulations

#Go= Go ,  SStop= Successful Stop,  FStop= Failed Stop#

#First: Check the fist stop trial#

if (GORT[1] > SSRT[1]+SSD[1]) { TrialType[1]<- ’SStop’ } else  { TrialType[1]<- ’FStop’ }

if (GORT[1] > SSRT[1]+SSD[1]) { Inhibition[1]<- ’1’ } else  { Inhibition[1]<- ’0’ }

if (GORT[1] > SSRT[1]+SSD[1]) {  index[1]<-  +50 } else  { index[1]<- -50}

if (GORT[1] > SSRT[1]+SSD[1]) {  SSD[2]<-  SSD[1]+50 } else  { SSD[2]<- SSD[1]-50}

#Second: Use information above to create a martingale of SSDs !#

for (i in 1:(m-1))

{

 if (index[i]== 50)  { SSD[i+1]<- SSD[i]+50 }

 if (index[i]== 50)  {if (GORT[i+1] > SSRT[i+1]+SSD[i+1]) { TrialType[i+1]<- ’SStop’ }

  else  {TrialType[i+1]<- ’FStop’}  }

 if (index[i]== 50)  {if (GORT[i+1] > SSRT[i+1]+SSD[i+1]) {  index[i+1]<- +50        }

  else  { index[i+1]<- -50       }  }

 if (index[i]== -50) { SSD[i+1]<- SSD[i]-50 }

 if (index[i]== -50) { if (GORT[i+1] > SSRT[i+1]+SSD[i+1]) { TrialType[i+1]<- ’SStop’ }

  else  {TrialType[i+1]<- ’FStop’  } }

 if (index[i]== -50) { if (GORT[i+1] > SSRT[i+1]+SSD[i+1]) {  index[i+1]<- +50        }

  else  { index[i+1]<- -50         } }

 if (index[i]== 50)  {if (GORT[i+1] > SSRT[i+1]+SSD[i+1]) { Inhibition[i+1]<- ’1’}

 else  {Inhibition[i+1]<- ’0’} }

 if (index[i]== -50) {if (GORT[i+1] > SSRT[i+1]+SSD[i+1]) { Inhibition[i+1]<- ’1’}

 else  {Inhibition[i+1]<- ’0’} }

}

#Third: Update Final Values of SSD given values of Index !#

SSRTSSD <- as.vector(matrix(0,nrow=1,ncol =n))

for (i in 1:n)

{

SSRTSSD[i] <- SSRT[i] + SSD[i]

}

#Fourth: We clear unwanted data for Go trials !#

for (i in (m+1):n)

{

  if (SSRT[i]!= ’NA’) {SSRT[i]= -999}

  if (SSRTSSD[i]!= ’NA’) {SSRTSSD[i]= -999}

  if (SSD[i] != ’NA’)  {SSD[i]= -999}

  if (index[i] != ’NA’) {index[i]= -999}

}

SRRT<-ifelse(TrialType %in% c(’FStop’), GORT, SRRT0)

GORT<-ifelse(TrialType %in% c(’SStop’,’FStop’), -999, GORT)

MAT=matrix(c(id,GORT,SSRT,SRRT,SSD,SSRTSSD,index,TrialType,Inhibition),nrow=length(GORT))

#Fifth: Combine to get the simulated SST data:

MATT<-MAT[sample(nrow(MAT)),]

colnames(MATT)<-c(’id’,’GORT’,’SSRT’,’SRRT’,’SSD’,’SSRTSSD’,’index’,’Trial’,’Inhibition’)

head(MATT)

write.csv(MATT,"Simulated_SST_Data_Tracking_Method.csv",row.names = F)

###End

## Appendix B. Cluster Type Weight Calculations in the Simulation of SST Data

Here we show that the weight *W_A_* = 0.75 is the most natural weight given independence of assignment of stop or go process to the given trial. To see this, let *T*_1_, ⋯, *T*_96_, *S*_1_, ⋯, *S*_24_ and *G*_1_, ⋯, *G*_72_ denote all trials, stop trials and go trials, respectively. Then, given that 75% of all trials are go trials, it follows that (A similar argument shows that, in general and conditioned on independence of assignment of stop or go process to the given trial, for given stop-signal probability *p_ss_* we have *W_B_* = *p_ss_*).

$$\begin{array}{ccc} W_{A} & = & \frac{\#\{\exists j\left(T_{i}=S_{j}\right),\exists k\left(T_{i-1}=G_{k}\right)|1\leq i\leq 96\}}{24}\\ & = & \frac{\#\{\exists j\left(T_{i}=S_{j}\right),\exists k\left(T_{i-1}=G_{k}\right)|1\leq i\leq 96\}/96}{24/96}\\ & = & \frac{P(\exists j\left(T_{i}=S_{j}\right),\exists k\left(T_{i-1}=G_{k}\right)|1\leq i\leq 96)}{P(\exists j\left(T_{i}=S_{j}\right)|1\leq i\leq 96)}\\ & = & \frac{P\left(\exists j\left(T_{i}=S_{j}\right)|1\leq i\leq 96\right)\times P\left(\exists k\left(T_{i-1}=G_{k}\right)|1\leq i\leq 96\right)}{P(\exists j\left(T_{i}=S_{j}\right)|1\leq i\leq 96)}\\ & = & P(\exists k\left(T_{i-1}=G_{k}\right)|1\leq i\leq 96)≃0.75,\\ W_{B} & = & 1-W_{A}=0.25. \end{array}$$
